# Quantification of Iris Concavity

**Published:** 2010-07

**Authors:** Rouzbeh Amini, Julie E whitcomb, Tiago S Prata, Syril Dorairaj, Jeffrey M Liebmann, Robert Ritch, Victor H Barocas

**Affiliations:** 1 Department of Biomedical Engineering, University of Minnesota, Minneapolis, Minnesota, USA; 2 Department of Mechanical Engineering, University of Minnesota, Minneapolis, Minnesota, USA; 3 Einhorn Clinical Research Center, New York Eye and Ear Infirmary, New York, New York, USA; 4 New York University School of Medicine, New York, New York, USA; 5 Department of Ophthalmology, New York Medical College, Valhalla, New York, USA

**Dear Editor,**

Iris contour is relevant to a number of clinical situations including primary angle-closure (pupillary block and plateau iris) and pigment dispersion syndrome. Since iris contour is known to be affected by various phenomena such as prevention of blinking^1^, exercise^2^ and accomodation^3^,^4^, a method is needed to quantify the shape of the iris and allow comparison among individuals or under different conditions (e.g. before and after accommodation). The most commonly applied method is iris concavity as defined by Liebmann et al^5^ and illustrated in figure 1. A chord is drawn from the iris root to the posterior margin of the pupil. Next, the longest possible perpendicular line is drawn from the chord to the posterior iris surface, and the length of the perpendicular is deemed as concavity. By convention, positive concavity indicates anterior bowing whereas negative concavity reflects posterior bowing.

While iris concavity is an easy measurement to make and provides the necessary basis for comparing iris contours, it suffers from significant drawbacks. Since this measurement deals with units of length, it is dependent on an accurate conversion from pixels to distance, which becomes especially important when images obtained by different modalities (e.g., OCT vs. ultrasound) are to be compared. An additional consequence is that images with the same shape but different size yield different measures (Fig. 1). Finally, because only the length of the perpendicular is considered, contours with different curvatures can generate the same concavity.

A convenient alternative would be the mathematical definition of curvature, namely the inverse of the radius of the circle passing through three points along the contour. This definition would eliminate some of the problems, but still remains a scale-dependent measurement (units of inverse distance). We propose the best choice to be the ratio of the perpendicular length (defining concavity in current methods) to chord length. This ratio, which we define as the *concavity ratio* (CR), is independent of scale and thus holds no bias for large versus small iris sizes. An additional benefit is that CR can be calculated directly from the image without calibration of pixel size.

An example of the proposed parameter is presented in figure 2, which shows OCT images of a 56-year-old man who received laser peripheral iridotomy for a narrow angle. The image taken in the dark (Fig. 2b) shows a more sharply curved iris but measures roughly the same concavity (15.4 vs. 15.0 pixels, a difference of only 3%) compared to the image taken under light conditions (Fig. 2a). When CR is calculated by taking the ratio of concavity to chord length, the difference in shape emerges, with a CR of 0.063 for figure 2a and 0.082 for figure 2b, reflecting a difference of 30%.

If one’s intent were to quantify displacement, iris concavity would be the most appropriate measure; however both images display similar amounts of anterior bowing and the displacement from linearity in the posterior iris surface is the same. If, however, one’s intent is to quantify changes in shape, then a scale-independent measure of shape such as CR should be used.

## Figures and Tables

**Figure 1:**
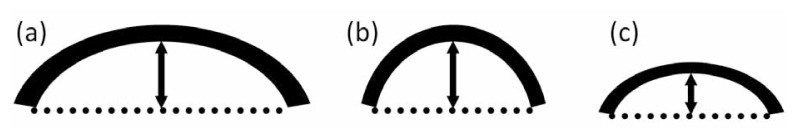
Iris concavity. The chord length (dotted line) is the distance from the iris root to the pupil margin. Concavity (arrows) is the longest distance from the chord to the posterior surface of the iris. Curves (a) and (b) have the same concavity even though (b) clearly has more pronounced curvature. Curves (a) and (c) are of identical shape except that (c) is smaller, resulting in a lower concavity despite comparable curvature. The ratio of concavity to chord length is 0.27 for (a) and (c), and 0.44 for (b).

**Figure 2:**
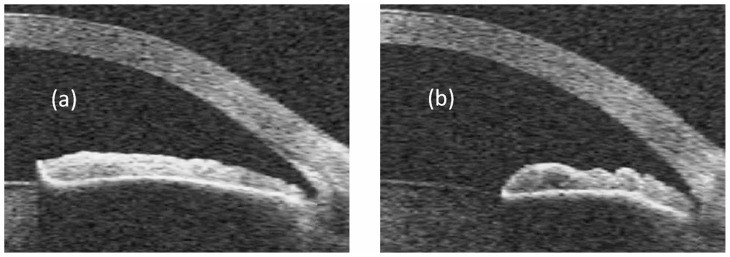
OCT images of the iris before and after dilation. **(a)** Under light conditions, when the pupil is constricted, the iris is bowed slightly to the anterior. **(b)** Under dark conditions, when the pupil is dilated, the iris becomes more sharply curved, but the concavity changes very little, since the main change is in the chord length. The ratio of concavity to chord length, however, increases by 30% (details in text).
